# Mean Propulsive Velocity Is a Viable Method for Adjusting the Resistance-Training Load at Moderate Altitude

**DOI:** 10.3389/fspor.2019.00052

**Published:** 2019-10-24

**Authors:** Lara Rodríguez-Zamora, Paulino Padial, Brad Jon Schoenfeld, Belén Feriche

**Affiliations:** ^1^Division of Sport Sciences, School of Health and Medical Sciences, Örebro University, Örebro, Sweden; ^2^Environmental Physiology Group, Department of Health Sciences, Mid Sweden University, Östersund, Sweden; ^3^Department of Physical Education and Sport, Faculty of Sport Sciences, University of Granada, Granada, Spain; ^4^Department of Health Sciences, Lehman College, New York, NY, United States

**Keywords:** hypobaric hypoxia, monitoring, power, resistance training, strength

## Abstract

We examined the viability of using mean propulsive velocity (MPV) to adjust the load in the countermovement jump (CMJ) at moderate altitude. Twenty-four volunteers were assigned to a 4-week power-oriented resistance training (R_T_) program in either normoxia (N, 690 m) or intermittent hypobaric hypoxia (IH, 2,320 m). The load was adjusted to maintain execution velocity of CMJ at 1m·s^−1^ of MPV. Relative peak power output (P_rel_), and percentage of velocity loss throughout the sets (VL) were determined for each session. The internal load was measured by the rating of perceived exertion (RPE). The absolute load lifted was higher in IH compared to N (75.6 ± 8.4 vs. 58.5 ± 12.3 kg *P* < 0.001). However, similar relative increases for both groups were found when comparing the final values (IH: 8.2%, *P* = 0.007; N: 9.8%, *P* = 0.03) with no changes in VL between groups (*P* = 0.36). Post-study P_rel_ improved significantly only in IH (+7% W·kg^−1^, *P* = 0.002). Mean RPE was greater in IH vs. N (6.8 ± 1.5 vs. 5.6 ± 2, *P* < 0.001). The MPV seems to be a viable method for adjusting external load during R_T_ at moderate altitude. However, given that R_T_ at moderate altitude increases RPE, it is prudent to monitor internal load when using the MPV to best determine the actual physiological stress of the session.

## Introduction

Altitude training (usually at moderate altitudes of 1,800–2,500 m) is a strategy widely used by athletes to improve performance at sea level. In terms of resistance training (R_T_), it has been found that hypoxia elicits specific adaptations, such us muscle plasticity, that modify the muscles' capacity to generate work resulting from a sensitivity to oxygen changes (Bosco et al., 2010). In addition, explosive actions seemingly benefit from hypobaric hypoxia exposure due to the decreased air resistance and modified motor unit recruitment patterns as a result of the increased anaerobic metabolism release (Scott et al., 2016b,c; Ramos-Campo et al., 2017). To this end, improvements in sprinting, throwing, and jumping performance at altitude have been reported after exposure to hypobaric hypoxia (Hamlin et al., 2015). This may be attributable not only to the mechanisms mentioned above, but also to the increased spinal excitability in acute exposure to hypoxia (Lundby et al., 2009), by which continuous exposure would not be needed to achieve muscle power improvements (Morales-Artacho et al., 2018). Accordingly, it has been shown that both acute and prolonged exposure to moderate altitude improved maximal power, movement velocity, and jump performance (Feriche et al., 2014; García-Ramos et al., 2016b).

On the other hand, a means to determine whether a R_T_ program is efficacious is by quantifying the associated stress imposed on the athlete (Scott et al., 2016a). When the training load is insufficient then adaptation might not occur, while excessive stress might impair performance and potentially increase injury risk (Halson, 2014). Hence, coaches record markers of internal [i.e., the athlete's individual responses, such as heart rate (HR) and ratings of perceived exertion (RPE)] and external (i.e., the work completed by the athlete, in terms of variables such as velocity, applied strength, and power output) loads to quantify the training stress and hence gauge the training efficacy (McLaren et al., 2017). In an attempt to integrate both the training session volume and intensity as a single variable, the use of session ratings of perceived exertion (sRPE × training time in min) provide a valid and reliable measure of the internal training load (Day et al., 2004; Sweet et al., 2004). A benefit of this strategy is that RPE considers the actual loads being lifted in concert with the number of repetitions, inter-set rest periods, and velocity of repetitions during the session (Scott et al., 2016a). Alternatively, several studies have indicated a close relationship between training intensity and the velocity achieved against a given absolute load, and the strength responses (González-Badillo et al., 2011, 2014). González-Badillo and Sánchez-Medina (2010) introduced the concept of the mean propulsive velocity (MPV) as an alternative way to prescribe loading intensity to the 1RM by using the movement velocity. The authors justified its use based on the fact that the actual velocity performed in each repetition could perhaps be the best reference to gauge the real effort incurred by the athlete. Consequently many coaches and athletes have embraced “*velocity-based training*” as a strategy for adjusting the intensity of the R_T_ programs. However, during R_T_ in isoinertial conditions, and assuming that with every repetition performed with maximal voluntary effort, velocity unintentionally declines as fatigue develops (Izquierdo et al., 2006). In fact, strong correlations were found between mechanical [velocity and countermovement jump (CMJ) height losses] and metabolic (lactate, ammonia) measures of fatigue supporting the validity of using velocity loss (VL) to objectively quantify neuromuscular fatigue during R_T_ (Sánchez-Medina and González-Badillo, 2011).

Environmental conditions, such as altitude, could produce neuromuscular fatigue during exercise by decreasing the force or power capacity (Amann and Calbet, 2008) as well as the muscle fibers' capacity for relaxation (Allen et al., 2008), Indeed, the reduction of oxygen delivery to the working muscles in hypoxia could exacerbate fatigue by impairing neuromuscular transmission during contractions (Amann et al., 2006). As a result, the same exercise carried out at altitude could be physiologically more demanding than at sea level so that higher altitudes induce a higher fatigue. Given these results and the aforementioned effects of hypoxia on muscle performance, it seems plausible that the force-velocity relationship at altitude could be affected by environmental conditions, making the adjustment of the load via MPV different than at sea level. Thus, the principal aim of this study was to examine if MPV is a viable tool to adjust the individual load during a power-oriented R_T_ program at moderate altitude. We hypothesized that similar load and VL patterns would be seen with the load properly adjusted in both groups.

## Materials and Methods

The present study used a longitudinal design with two parallel groups [living and training in normoxia (N; *n* = 11), and living in N and training at intermittent moderate altitude (IH; 2,320 m); *n* = 13] to compare the changes in variables linked to a 1 m·s^−1^ of MPV (Loturco et al., 2015) [load lifted (kg) and peak power output (W)–both in absolute and relative values, and the percentage of velocity loss throughout the sets (%, VL)] when executing a countermovement jump (CMJ) during a 4-week power-oriented R_T_ program. The internal load was measured by the RPE (Morree et al., 2012). Subjects performed two R_T_ sessions · week^−1^ (eight in total) under the two different conditions. The subjects were assigned to each group according to their availability, making this a quasi-experimental design. The IH group conducted the training sessions at the High-Performance Center in Sierra Nevada (2,320 m), while the N group trained at the Faculty of Sport Sciences laboratory (690 m). In order to avoid injuries and to standardize technique before the R_T_ program, all of the volunteers took part in a pre-intervention 4-week conditioning-training program. At the beginning of the study there were no significant differences between groups in terms of absolute load lifted (IH: 68.0 kg vs. N: 60.5 kg, *P* > 0.05) and P_rel_ at 1 m·s^−1^ of the MPV (IH: 47.2 W·kg^−1^ vs. N: 43.9 W·kg^−1^, *P* > 0.05), tested in normoxia. To minimize the potential for instruction bias, the testers remained the same for both groups.

### Subjects

Twenty-four collegiate-men with at least 2 years of R_T_ experience volunteered for the study. The mean ± SD of age, height, body mass, BMI, fat mass, and fat free mass were; 23.0 ± 3.3 years, 177.8 ± 6.9 cm, 75.9 ± 8.5 kg, 23.9 ± 1.6 kg·m^−1^, 13.4 ± 3.2 kg, and 45.0 ± 4.2 kg, respectively. The inclusion criteria were: not having been exposed to more than 3–4 days of altitudes in excess of 1,500 m within 2 months before the study; no chronic diseases or recent musculoskeletal injuries, and not currently using drugs/ergogenic aids that could impact muscular function. Subjects received oral and written information before giving their informed consent. This study was approved by the local Research Ethics Committee and conducted in accordance with the Helsinki Declaration.

### Procedures

#### Pre-intervention

After a general and specific warm-up (5 min jogging, joint mobility exercises, and five unloaded CMJ), subjects completed a CMJ test using the following loads under normoxic conditions: 17, 30, 45, 60, and 75 kg (Morales-Artacho et al., 2018). Two attempts were performed for each load with a 1 min pause between repetitions and a 3 min pause between loads (Morales-Artacho et al., 2018). Subjects were instructed to perform the CMJ with ~90° of knee flexion, and to jump as high as possible. For every repetition the MPV was recorded and the repetition with the highest MPV was selected for further analysis. Fifteen minutes after the test, subjects carried out a 15-repetition CMJ test at maximum intended velocity (Morales-Artacho et al., 2018). The external load used during the test was individually selected as the load linked with the barbell MPV of 1 m·s^−1^, interpolated from the load-velocity relationship achieved on that day. The average P_rel_ of the set was used for further comparisons.

#### Training Session

**Figure 1:**
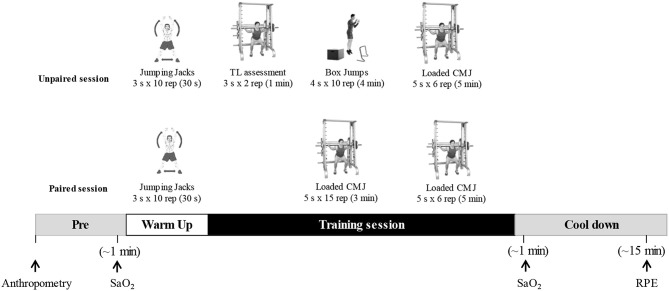
Training session with anthropometry, arterial oxygen saturation (SaO_2_), and rating of perceived exertion (RPE) assessment. TL, training load; CMJ, Countermovement jump. For the training session sets, repetitions and resting periods are shown as follows: sets × repetitions (inter-set recovery).

Subjects performed 4 weeks (two sessions · week^−1^) of a power-oriented training program (Figure 1) (Morales-Artacho et al., 2018). Each session began with a 15 min warm up (5 min of aerobic activity, 5 min of lower-body mobility exercises, and three sets of 10 repetitions of jumping jacks). After the warm-up, five sets of six loaded CMJs were performed with a load linked to 1 m·s^−1^ of the MPV, each with a 5 min inter-set rest period. This load-velocity ratio is considered optimal for muscle power training using CMJ exercise and corresponds to 50–55% of 1 repetition maximum (Pérez-Castilla et al., 2018). Subjects jumped as high as possible after performing a countermovement jump to a self-selected depth of ~90° knee flexion (Jiménez-Reyes et al., 2017). In addition, on the first day of every week (unpaired session), the loaded CMJ was preceded by four sets of 10 box jumps at different heights and depths with a 4 min rest, while on the second day (paired session), it was preceded by five sets of 15 CMJs followed by a 3 min rest between sets executed under the same conditions as the loaded CMJ.

#### External Training Load Adjustment

The load displaced at a velocity of 1 m·s^−1^ of the MPV was assessed weekly during the training program. For this determination, subjects performed three sets of two CMJs after the warmup of the first training session of the week. The initial load was set at 20 kg for all subjects and then progressively increased three times (by 15–20 kg each time) until the MPV was lower than 0.9 m·s^−1^. For every increment, two attempts were executed with a 1 min inter-rep rest and a 3 min rest between load changes. The exact load linked to a 1 m·s^−1^ of the MPV was calculated by interpolation from the individual load-MPV linear regression equation.

#### Measurements

##### External load markers

All loaded CMJs were performed using a Smith Machine (Technogym, Barcelona, Spain). MPV data was obtained from a linear velocity transducer (T-Force System; Ergotech, Murcia, Spain). The dynamic measurement system was fixed perpendicular to the bar with a tether, reporting the vertical velocity at 1000 Hz. The variables were: (1) the displaced load (in kg at 1 m·s^−1^ of the MPV); (2) the maximal relative power achieved during the concentric phase of the movement (W·kg^−1^, P_rel_), and; (3) the percentage of velocity loss (%, VL), defined as the change in average peak velocity during the execution of the first and second rep vs. the fifth and sixth rep during the same loaded CMJ set. The daily VL average during the whole R_T_ program was calculated and differences between the first session compared to the others (from two to eight) were used for comparisons.

##### Arterial oxygen saturation

Immediately before and after each testing session, arterial oxygen saturation (SaO_2_) was measured per duplicate using a pulse oximeter (Wristox 3100; Nonin, Plymouth, MN, USA). SaO_2_ values were calculated as the average for every 5 s period. The averaged pre- and -post-session SaO_2_ values were considered for further analysis.

##### Rating of perceived exertion

The RPE was rated ~15 min after the session using the Borg CR-10 scale (Foster et al., 2001). To ensure the quality of the data collected, all subjects were instructed on the use of the CR-10 scale 1 week prior to starting the training protocol. In addition, their usage of the CR-10 scale was monitored during this week on three separate sessions (Psycharakis, 2011). Given that all sessions had the same duration, the RPE value, instead of the session-RPE, was considered for further analysis.

#### Statistical Analysis

Data are presented as means and standard deviations (SD). Normality was assessed using the Shapiro-Wilk's test. A three-factor mixed model ANOVA with a between-subject factor (N *vs*. IH) and two within-subject factors [(session: from the first to eighth) and time (pre-post training session)] were applied on SaO_2_. Another ANOVA with one between-subject factor (N vs. IH) and one within-subject factor (session: from the first to eighth) was used for the load, P_rel_ and VL comparisons. A two factor ANOVA was used to evaluate the effect of the environmental conditions (N vs. IH) and the time (pre-post session) on the variables: load and P_rel_. The within-subject effect was determined using the Greenhouse-Geisser test or the Huynh-Feldt correction for degrees of freedom in cases where the result of the Mauchly sphericity test was significant. Bonferroni *post-hoc* tests was used for multiple pairwise comparisons. Eta squared ($\eta_{p}^{2}$) for main effects were calculated for the ANOVAs, where the values of the effect sizes were considered as follows: 0.02 (small), 0.13 (medium), and 0.26 (large) (Bakeman, 2005). Friedman and Mann-Whitney U tests were used to analyze changes in the RPE. Pearson correlations were employed to quantify the association between markers of external load and the VL for each group. The level of significance was set *a priori* at *P* < 0.05. Statistical analyses were conducted using SPSS Statistics for Windows (v. 22; IBM Corp., Armonk, NY).

## Results

### External Load Markers

**Figure 2:**
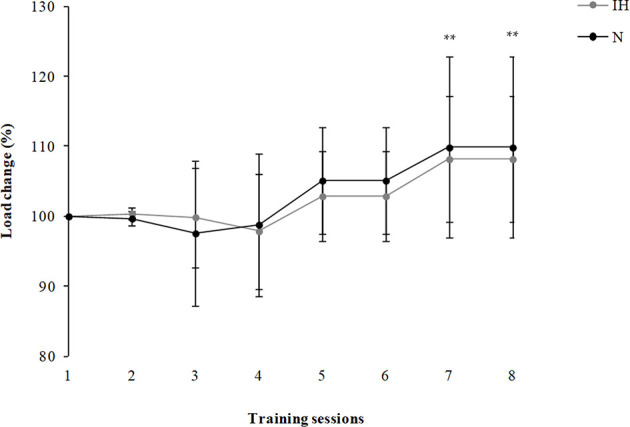
Change in the training load that elicits 1 m·s^−1^ of the mean propulsive velocity (MPV) expressed in percentage (%) for the 8 sessions of the resistance training program. ^**^Significant differences among sessions (compared with the first session, *P* < 0.05) were noted for both groups.

On average, the absolute load lifted was 22.6% higher in the IH group compared to the N group (Table 1) with 79.8 ± 9.3 kg and 62.2 ± 13.9 kg being the final absolute load achieved in their respective conditions. However, similar relative increases in this variable were found for both groups when comparing the final values (IH: 8.2%, *P* = 0.007; N: 9.8%, *P* = 0.03; Figure 2).

**Table 1:** Mean values of external and internal training load markers per group.

	**Load markers**	**Groups**	
		**IH (*n* = 11)**	**N (*n* = 13)**
External	Absolute displaced load (kg)	75.6 ± 8.4^#^	58.5 ± 12.3
	Relative displaced load (kg·kg^−1^)	1.0 ± 0.1^#^	0.8 ± 0.2
	Absolute power (W)	3,580.5 ± 394.5^#^	3,261.5 ± 492.4
	Relative power (W·kg^−1^)	46.9 ± 4.5^#^	42.7 ± 4.8
Internal	RPE (a.u)	6.8 ± 1.5^#^	5.6 ± 2.1

*Data are mean ± SD (n = 24) for the 4-weeks R_T_ program. IH, intermittent hypoxia group; N, normoxia group. Significance #(P < 0.001)*.

**Figure 3:**
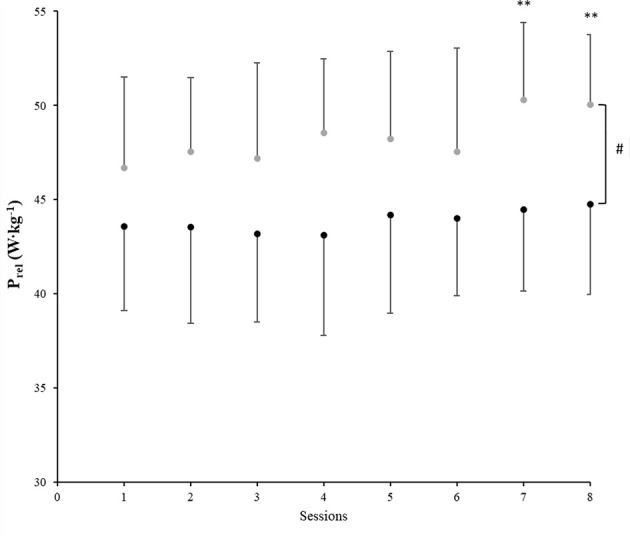
Session averaged relative peak power (P_rel_) ± SD during the 5 × 6 repetitions-set exercises for both groups (IH: 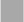, N: 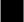), across the 4-weeks R_T_ program. Significant differences were ^**^among sessions (compared with the first session, *P* < 0.001) and ^#^between groups (*P* < 0.05).

In regard to power output, higher values of both absolute and relative values were also found in the IH group compared to the N group (Table 1) with the IH group achieving 50.1 ± 3.7 W·kg^−1^ in the last training session while the N group achieved 44.8 ± 4.8 W·kg^−1^ (*P* = 0.007). Furthermore, in the IH group improvements in P_rel_ also displayed a significant “session” main effect (*F* = 6.3, *P* < 0.001, $\eta_{p}^{2}$ = 0.2) by increasing significantly at the end of the R_T_ program when compared to the first session (vs. seventh and eight; *P* < 0.001, Figure 3). The improvement in P_rel_ was also reached faster in IH (from session 1 vs. 4 and following; *P* < 0.05) compared to N [at session 1 vs. 8, *P* = 0.19; 95%IC (−3.2; −0.4)]. When the pre-post comparison in terms of P_rel_, both groups displayed improvements in this variable (N: 3.7% W·kg^−1^ and IH: 7.0% W·kg^−1^); however, the improvement was only significant in the IH group [pre: 50.7 W·kg^−1^ vs. post: 54.1 W·kg^−1^; *P* = 0.002).

For intra session VL, no differences between groups (*F* = 0.9, *P* = 0.36, $\eta_{p}^{2}$ = 0.04; *F* = 0.14) or “session” (*P* = 0.34, $\eta_{p}^{2}$ = 0.05) were detected throughout the R_T_ period.

### Arterial Oxygen Saturation

The average SaO_2_ values were significantly lower in IH compared to N, both before (93.6 ± 2.1 vs. 97.7 ± 0.9%; *F* = 47.8, *P* < 0.001, $\eta_{p}^{2}$ = 0.7) and after the program (93.6 ± 1.5 vs. 97.0 ± 0.8%; *F* = 78.7, *P* < 0.001, $\eta_{p}^{2}$ = 0.8). Significant differences between pre and post session SaO_2_ values were found only in N (*F* = 22.0, *P* = 0.001, $\eta_{p}^{2}$ = 0.7), with lower values observed after the session.

### Rating of Perceived Exertion

**Figure 4:**
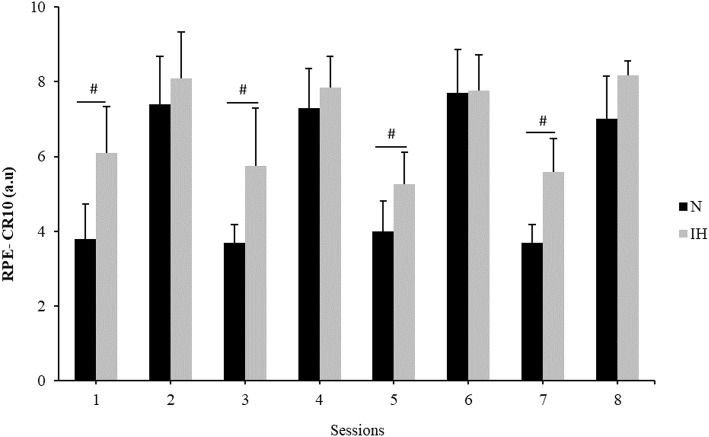
RPE-CR10 (a.u) scores of both groups (IH: 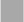, N: 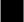) for each training session. ^#^Significant differences (*P* < 0.05) were between groups.

Table 1 shows the mean RPE per group. Greater RPE values were observed in IH when compared with N for all the unpaired sessions (6.8 ± 1.5 vs. 5.6 ± 2 a.u, *P* < 0.001, respectively; Figure 4).

## Discussion

The aim of this study was to examine the viability of the MPV to adjust the individual load during a power-oriented R_T_ program at moderate altitude. The similar response observed between the groups in terms of relative load (Figure 2), combined with the lack of differences in VL between groups supports the use of MPV as a viable strategy to adjust the external training load at moderate altitude, as previously tested at sea level (Izquierdo et al., 2006; González-Badillo and Sánchez-Medina, 2010). On the other hand, the higher RPE values and the greater displaced load seen in the IH group suggest that, “*velocity-based training*” at moderate altitude increases both the internal and the external load. Therefore, it seems that in hypoxia the physiological stress imposed by the training session at 1 m/s is higher than in normoxia. For this reason, it is recommended to also monitor the internal load when using the MPV; in this way, coaches are more informed as to the real physiological stress associated with the R_T_ session. It is worth mentioning that despite the training effect, higher P_rel_ values and faster improvements in this parameter were reached in the IH group when compared to N. This could be interpreted as an increased effectiveness of the R_T_ at altitude.

It is well-known that exercise in hypoxia relies on a greater contribution from the anaerobic energy systems, accelerating the production of metabolites, which in turn may have positive effects on the strength responses due to an enhanced muscle activation (Kawada, 2005; Schoenfeld, 2013). In addition, several studies suggest that low levels of SaO_2_ can induce the recruitment of additional type II fibers (Kon et al., 2010; Schoenfeld, 2013). Manimmanakorn et al. (2013) observed that breathing air with low O_2_ content is a primary trigger for muscle fiber transition (from type I to II), making the movement faster because of the larger motor neurons' capability to conduct impulses at higher velocities (Manimmanakorn et al., 2013). Thus, at altitude, the P_rel_ production could be also related to the recruitment of more fast-twitch fibers (type II), which are primarily glycolytic, playing an important role for the energy supply during R_T_ (Maffiuletti et al., 2016). However, during isolated explosive movements, the hypoxic benefits on performance have been found only in hypobaric hypoxia and not in normobaric hypoxia (Feriche et al., 2014; Scott et al., 2015). Thus, despite the fact that both groups started the 4-week program in their corresponding environmental condition, having similar levels of displaced load (first session, *N*: −3.7 Kg, *P* > 0.05; IH: +4.9 Kg; *P* = 0.046 with respect the pre-test in N conditions), it seems that the moderate altitude (2,300 m) characteristics (reduced resistance of the air and lower PaO_2_) combined with the muscle response to the hypoxic stimulus (an increased recruitment of high-threshold type II fibers) would have had a positive effect not only on the amount of weight displaced but also on the load-velocity relationship in IH (Feriche et al., 2014; García-Ramos et al., 2016a). This hypothesis is supported by the fact that, on average, the IH group showed lower values of SaO_2_ and higher values in all external load markers (Table 1).

Our results lend support to the findings of other studies that reported increases in both maximal peak velocities and power output at the end of a power-oriented R_T_ period in chronic (García-Ramos et al., 2016b), or intermittent natural altitude (Morales-Artacho et al., 2018). Therefore, it seems likely that muscle function is not impaired by acute or chronic exposure to moderate altitude in comparison to normoxia (García-Ramos et al., 2016b), and it may in fact be enhanced after a training period at altitude (García-Ramos et al., 2016b; Morales-Artacho et al., 2018). Conversely, in a study in which subjects were exposed to normobaric hypoxia (breathing through a mask connected to a hypoxic generator), no differences were found regarding P_rel_ when executing CMJs in normoxia (FiO_2_ 21%), moderate hypoxia (FiO_2_ 16%) and “high altitude” (FiO_2_ 13%), probably due to all the conditions being tested under the same barometric pressure (Ramos-Campo et al., 2016).

As previously mentioned, monitoring the velocity loss can be used to determine the presence of neuromuscular fatigue with the concomitant impaired performance during R_T_ as an increase in this parameter is related to markers of metabolic stress (Sánchez-Medina and González-Badillo, 2011). The fact that we found a similar pattern in VL between groups during the R_T_ program (Figure 3) could be interpreted as evidence that muscular function did not deteriorate in the IH group. We had hypothesized that with the load properly adjusted (1 m·s^−1^ of MPV) for each individual session, the level of neuromuscular fatigue would be similar under both conditions; this hypothesis was confirmed.

On the other hand, RPE is one of the most common means of assessing internal load. It is known that athletes who exhibit a higher internal load to standardized external load may be losing fitness or suffering from fatigue (Impellizzeri et al., 2018). The combination of both internal and external load markers may indicate what kind of fatigue the athlete is suffering from Impellizzeri et al. (2018). While muscle fatigue increases the HR, RPE, and VL (Marcora et al., 2008; Sánchez-Medina and González-Badillo, 2011), mental fatigue increases only RPE (Marcora et al., 2009). Given that the IH group showed higher levels of RPE (Figure 4) with a similar VL pattern than N (Figure 2), it is plausible to speculate that the IH participants were more mentally fatigued. We hypothesized that hypoxia combined with exercise would have had a greater influence on RPE than those related to exercise in normoxia (Pandolf, 2001). It is well-known that RPE can be used to measure the effort during R_T_ (Gearhart et al., 2002), with perception purportedly related to metabolic stress markers and the magnitude of muscle activation (Lagally et al., 2002). When exercise is performed in normoxia and hypobaric hypoxia at the same relative intensity (1 m·s^−1^ of MPV), certain physiological responses are altered (e.g., reduction in SaO_2_), accounting for the increased RPE values in IH (Pandolf, 2001). The metabolic needs of the lower extremities can also increase RPE values because more muscle mass is at work (Marais et al., 2001). It therefore follows that the same muscle groups working in hypoxia would evoke higher RPE values. Our results are consistent with those of Ramos-Campo et al. (2016), who showed differences in RPE between normoxia (~sea level) and high altitude (~3,800 m) after a R_T_ circuit (Ramos-Campo et al., 2016).

Statistical analysis showed that sessions at IH during the unpaired days were considered harder than at sea level (Figure 4). Several studies have suggested that RPE during R_T_ may be more dependent on the intensity than on the set volume (Gearhart et al., 2002; Day et al., 2004) due to a greater fatigability of type II fibers. Sweet et al. (2004) reported a positive relationship between intensity and RPE during R_T_, whereby a high force or power output during the first part of the session conceivably fatigued the type II fibers so they were unable to perform over an extended period. Thus, high repetition protocols that engage a greater use of the type II fibers are perceived as harder than comparable intensities of steady-state aerobic exercise on a cycle ergometer (Sweet et al., 2004). Our results support this theory as the IH group who had higher RPE values also improved P_rel_ faster than N (Figure 3). Another factor that could have influenced the RPE during the unpaired sessions are the plyometric exercises (Figure 1). It is well-known that during plyometric exercises muscles are trained under tensions greater than those achieved by conventional resistance training (Holcomb et al., 1996), which in turn may increase the intensity of effort in the exercise performed. Given these results, we speculate that the combination of hypoxia and intense exercises may explain RPE during R_T_ at moderate altitude. However, this hypothesis requires further investigation utilizing a broad range of intensities and altitudes above the sea level.

A limitation of the present study is that the composition of both groups (N and IH) were determined by convenience (participants' availability) and not truly randomized. We therefore cannot rule out the possibility that inherent initial differences between N and IH may have been present that compromised internal validity. However, the lack of significant between-group differences before starting the 4-week R_T_ program, noted in any of the baseline parameters would seem to indicate this did not influence results. Monitoring another internal load parameter (e.g., HR, lactate, ammonia, creatinine…) together with the current RPE would have given us more information about the relationship between the internal and external load markers. This would have helped to better identify how the participants were coping with the external stimuli as a result of the MPV application.

In conclusion the present study demonstrated that 1 m·s^−1^ of MPV is a viable tool to adjust the individual load during power-oriented R_T_ at moderate altitude. While the aim of the present study was not focusing on the effectiveness of a “live low, train high” strategy applied to R_T_, the results revealed that the intermittent exposure to moderate altitude can be useful to enhance the relative peak power output more expeditiously than at sea level. However, the results indicate that power-oriented exercises at moderate altitude allow athletes to lift higher loads, evoking higher levels of RPE than at sea level. This suggests that the physiological stress imposed by a training session at 1 m/s is higher in hypoxia compared to normoxia. Therefore, even though the application of velocity-based training at moderate altitude may be superior for improving performance compared to a traditional system of %1RM, we recommend combing the use of the MPV with another internal marker to determine whether demands from the training session stimuli are appropriate.

## Data Availability Statement

The raw data supporting the conclusions of this manuscript will be made available by the authors, without undue reservation, to any qualified researcher.

## Ethics Statement

The studies involving human participants were reviewed and approved by Ethics committee on Human Research, Granada University. The patients/participants provided their written informed consent to participate in this study.

## Author Contributions

LR-Z, PP, and BF: conceived and designed the experiments and performed the experiments. LR-Z: analyzed the data and wrote the paper. BS: checked English spelling and grammar. BS and BF: revised the paper. All the authors have reviewed and approved the manuscript prior submission.

### Conflict of Interest

The authors declare that the research was conducted in the absence of any commercial or financial relationships that could be construed as a potential conflict of interest.
